# Incidence rates of allergy diagnoses in the U.S. military health system after the COVID-19 pandemic

**DOI:** 10.3389/falgy.2026.1884771

**Published:** 2026-08-21

**Authors:** J. Joseph Brough, Zella Berill, Apryl Susi, Elizabeth Paloma Sanchez, David Schwartz, Cade M. Nylund, Edward Mitre

**Affiliations:** 1 Department of Internal Medicine, Walter Reed National Military Medical Center, Bethesda, MD, United States; 2 Henry M. Jackson Foundation for the Advancement of Military Medicine, Inc., Bethesda, MD, United States; 3 Department of Pediatrics, Uniformed Services University of the Health Sciences, Bethesda, MD, United States; 4 Department of Microbiology and Immunology, Uniformed Services University of the Health Sciences, Bethesda, MD, United States; 5 Laboratory of Allergic Diseases, National Institute of Allergy & Infectious Diseases, Bethesda, MD, United States

**Keywords:** allergy diagnoses, COVID-19 pandemic, hygiene hypothesis, infection—immunology, public health policy

## Abstract

**Introduction:**

During the COVID-19 pandemic, exposures to environmental allergens and microorganisms were dramatically altered, potentially impacting the development and diagnosis of allergic conditions.

**Methods:**

We conducted a retrospective, repeated, monthly cross-sectional study examining monthly incidence rates of allergic diseases diagnosed before and throughout the first 3 years of the COVID-19 pandemic in individuals aged 0–64 years enrolled in the TRICARE Management Activity Military Health System (MHS) Data Repository (MDR). Allergic diseases were grouped into twelve categories. Rates of incident outcomes were calculated monthly and compared between subgroups and during the pre-pandemic year (March 1, 2019–February 29, 2020) and the first 3 years after the COVID-19 Pandemic beginning on March 1, 2020.

**Results:**

Allergy diagnoses of all twelve conditions assessed were lower during COVID-19 year 1 (incidence 430 per 100,000) compared with the pre-pandemic year (incidence 750 per 100,000). This reduction in allergic diagnoses during COVID-19 year 1 was statistically significant for all allergic diagnoses queried. During COVID-19 years 2 and 3, allergy diagnoses of all conditions, except for anaphylaxis and food protein induced enterocolitis syndrome (FPIES) remained lower compared with the pre-pandemic year. The decrease in allergic diagnoses was greater than the decrease in total MHS healthcare visits.

**Discussion:**

In the MHS, for the first 3 years of the COVID-19 pandemic, allergic diagnoses were lower than in year prior to the pandemic. This reduction cannot be fully explained by decreased healthcare utilization.

## Introduction

Human behavior and exposures to microbes and allergens have long been postulated to significantly impact both the development and the course of allergic conditions (1, 2). Studies have shown an increased incidence of asthma in infants born via C-section compared to those delivered vaginally, likely due to differences in bacterial exposures (3). Other studies show an inverse relationship between the diversity of gut microbiota and atopic conditions such as asthma, possibly due to conditioning of the human respiratory tract and immune system by diverse microbiologic exposures (1). During the first years of the COVID-19 pandemic, human behavior was substantially altered through social distancing programs, government-mandated stay-at-home orders, and near universal masking (4). These behavioral changes likely changed the degree of exposures to both environmental allergens and microorganisms.

The aim of this study was to determine the potential impact of the COVID-19 pandemic on the incidence of allergic diseases through a large scale, population-based cohort analysis. To accomplish this, we analyzed allergy diagnosis incidence rates before and then during the first 3 years of the COVID-19 pandemic in individuals aged 0–64 years who were enrolled in the United States (U.S.) Military Health System (MHS). The MHS is a large, world-wide cohort that had an annual enrollment of over 7 million individuals in the study's eligible age range during the study period. Additionally, because environmental factors that influence allergy outcomes have been postulated to be most impactful during infancy (1, 2), this study also conducted a sub-analysis on allergy diagnoses for 3-year-olds.

## Methods

### Study design and population

We conducted a retrospective, repeated, monthly cross-sectional study examining monthly incidence rates of allergic diseases diagnosed before and throughout the first 3 years of the COVID-19 pandemic in individuals enrolled in the TRICARE Management Activity Military Health System (MHS) Data Repository (MDR). The population of the MHS includes active duty servicemembers, retirees and their dependents who receive routine care at both military clinics and treatment facilities as well as at civilian (purchased care) facilities. For these individuals, the database includes all outpatient and inpatient billing records as well as outpatient pharmacy use in both military and civilian facilities.

### Data acquisition and case definition

Data from the MDR was queried for all beneficiaries 0–64 years of age from January 2018 through February 2023. Allergic diseases were defined using the International Classification of Diseases, Tenth Revision (ICD-10) diagnostic codes and grouped into twelve categories: allergic rhinoconjunctivitis, asthma, medication allergy, atopic dermatitis, urticaria, food allergies, contact dermatitis, insect allergy, anaphylaxis, eosinophilic diseases, food protein induced enterocolitis syndrome (FPIES) and other allergy (Supplementary Table 1). We used an “any allergy” umbrella category to categorize all people who received a diagnosis in any of these twelve categories. Incident diagnoses were defined as ICD-10 codes not present for an individual from January 1, 2018 through February 28, 2019 and present for the first time for an individual on or after March 1, 2019. Incident diagnoses were tabulated monthly for both the entire cohort and for 3-year-olds at time of diagnosis. Demographic characteristics of age, geographic location, sex, and sponsor military rank were obtained from the Defense Enrollment Eligibility Reporting System (DEERS). Age groups were defined as <1 year old, 1–5 years old, 6–11 years old, 12–17 years old, 18–24 years old, 25–50 years old, and 51–64 years old. Geographic location was grouped into TRICARE regions used prior to January 1, 2025 of North, South, West, and overseas according to the DEERS defined categories of residence or duty location. Sponsor rank was categorized into junior enlisted, senior enlisted, and officer/other and used as a surrogate for education and income status. The study was divided into four periods. The “Pre-pandemic” period was defined as March 1, 2019–February 29, 2020. COVID-19 year 1 was defined as March 1, 2020–February 28, 2021, COVID-19 year 2 as March 1, 2021–February 28, 2022, and COVID-19 year 3 as March 1, 2022–February 28, 2023.

### Statistical analysis

All analyses were performed for both the full population and for a subset of 3-year-olds only. Descriptive statistics were generated with frequencies and percentages for categorical variables and medians and interquartile ranges (IQR) for continuous variables. Rates of incident outcomes were calculated monthly by taking the number of individuals with a new diagnosis in a given month divided by the number of beneficiaries enrolled in that same month. Because of fluctuations in MHS enrollment during the study period, these values were calculated on a month-by-month basis. For categorical variables, frequencies and percentages were calculated as the average monthly frequency or percentage over the study period. For continuous demographic variables, values were calculated as the mean of the monthly median and IQR recorded within each COVID-19 year. Incident rate ratios comparing COVID-19 years 1–3 with the pre-pandemic year were adjusted for age group, geographic location, sex, active-duty status, and sponsor military rank using models constructed with Bonferroni corrected Poisson regression. The Bonferroni correction was applied for the twelve allergic conditions studied, reducing the value of alpha for each condition from 0.05 to 0.0042 to account for multiple comparisons. All analyses were conducted using SAS version 9.4 (Cary, NC), and figures were created using the ggplot2 (vers 3.5.1, Wickham, 2016) package in R (vers. 4.4.1, R Core Team, 2024) (5, 6) and GraphPad Prism (GraphPad Prism version 10.6.1 for Windows, GraphPad Software, Boston, Massachusetts USA, https://www.graphpad.com).

## Results

The total population of 0–64 year old individuals in the MHS averaged between 7,102,350 (average population during COVID-19 year 3) to 7,264,494 (average population during COVID-19 year 1, Table 1). The population was 47% female (range 47.2%–47.4%), with a median age ranging from 28.0 to 28.9 years throughout the study. During the 4 years analyzed, the ranks of individuals remained fairly constant, with 60.0%–60.2% being senior enlisted, 17.0%–17.6% junior enlisted, and 22.4%–22.8% officers. Geographic areas of residency were also fairly constant throughout the 4 years, with 32.2%–32.8% of individuals living in the South region of the U.S., 30.9%–31.4% of individuals living in the North region of the U.S., 29.1%–29.5% living in the West, and 6.8%–7.2% living outside the continental U.S. (Table 1). Median ages of diagnosis for each of the 12 allergy categories are listed by year in Supplementary Table 2.

**Table 1:** Mean monthly demographic data breakdown by COVID-19 study period.

Participant characteristics	Pre COVID-19	COVID-19 year 1	COVID-19 year 2	COVID-19 year 3
Enrollment in MHS	7,242,994	7,264,494	7,234,749	7,102,350
Proportion female	47.4%	47.3%	47.2%	47.3%
Median (IQR) age	28.0 (16.0, 47.0)	28.0 (16.0, 46.8)	28.2 (16.0, 46.2)	28.9 (16.0, 46.0)
Proportion north	31.4%	31.4%	31.3%	30.9%
Proportion south	32.2%	32.3%	32.5%	32.8%
Proportion west	29.5%	29.5%	29.4%	29.1%
Proportion OCONUS	6.9%	6.8%	6.8%	7.2%
Proportion junior enlisted	17.2%	17.6%	17.6%	17.0%
Proportion senior enlisted	60.2%	60.0%	60.0%	60.2%
Proportion officer	22.4%	22.5%	22.5%	22.8%
Percentage 3-year-olds	1.6%	1.5%	1.5%	1.5%
Number 3-year-olds	114,651	111,320	108,903	105,223

IQR, interquartile (25% and 75%).

For the entire cohort, allergy diagnoses of all twelve conditions assessed were lower during COVID-19 year 1 compared with the pre-pandemic year (Table 2), with the monthly incident rate for any new allergy diagnosis decreasing from 750 per 100,000 people in the pre-pandemic year to 438 per 100,000 people in COVID-19 year 1. The four most diagnosed allergic diseases during the 4 years of observation were allergic rhinoconjunctivitis, asthma, medication allergy, and atopic dermatitis. In the pre-pandemic year, mean monthly incident rates for these diagnoses were 471, 158, 148, and 139 per 100,000 people, respectively (Table 2). By COVID-19 year 2, mean monthly incident rates of these four diagnoses were markedly decreased at 269, 91, 97, and 101 per 100,000 people, respectively. The incidence rates for all other atopic conditions are listed in Table 2.

**Table 2:** Mean monthly diagnoses and monthly incident rates (per 100,000 people) of allergic conditions, entire population.

Diagnosis	Pre-COVID-19	COVID-19 year 1	COVID-19 year 2	COVID-19 year 3
Any allergic condition	54,338 (750.32)	31,834 (438.22)	32,484 (449.00)	29,304 (412.21)
All. rhinoconjunctivitis	34,110 (471.03)	19,563 (269.31)	20,861 (288.39)	19,079 (268.19)
Asthma	11,445 (158.03)	6,613 (91.04)	6,891 (95.26)	7,101 (99.98)
Medication allergy	10,686 (147.54)	7,028 (96.75)	7,351 (101.59)	6,722 (94.62)
Atopic dermatitis	10,040 (138.63)	7,351 (101.19)	7,453 (103.05)	7,101 (99.96)
Other allergy	6,279 (86.70)	4,581 (63.05)	5,226 (72.23)	5,234 (73.66)
Urticaria	5,628 (77.70)	3,836 (52.80)	4,565 (63.10)	4,522 (63.65)
Food allergies	4,323 (59.68)	3,163 (43.54)	3,482 (48.13)	3,287 (46.28)
Contact dermatitis	3,951 (54.55)	3,172 (43.65)	3,124 (43.16)	2,879 (40.51)
Insect allergy	707 (9.76)	502 (6.91)	524 (7.24)	484 (6.81)
Anaphylaxis	296 (4.09)	241 (3.31)	316 (4.36)	362 (5.10)
FPIES	64 (0.88)	61 (0.84)	73 (1.01)	75 (1.05)
Eosinophilic disease	82 (1.13)	56 (0.77)	36 (0.49)	34 (0.47)

All. rhinoconjunctivitis, allergic rhinoconjunctivitis; FPIES, food protein induced enterocolitis syndrome.

**Figure 1:**
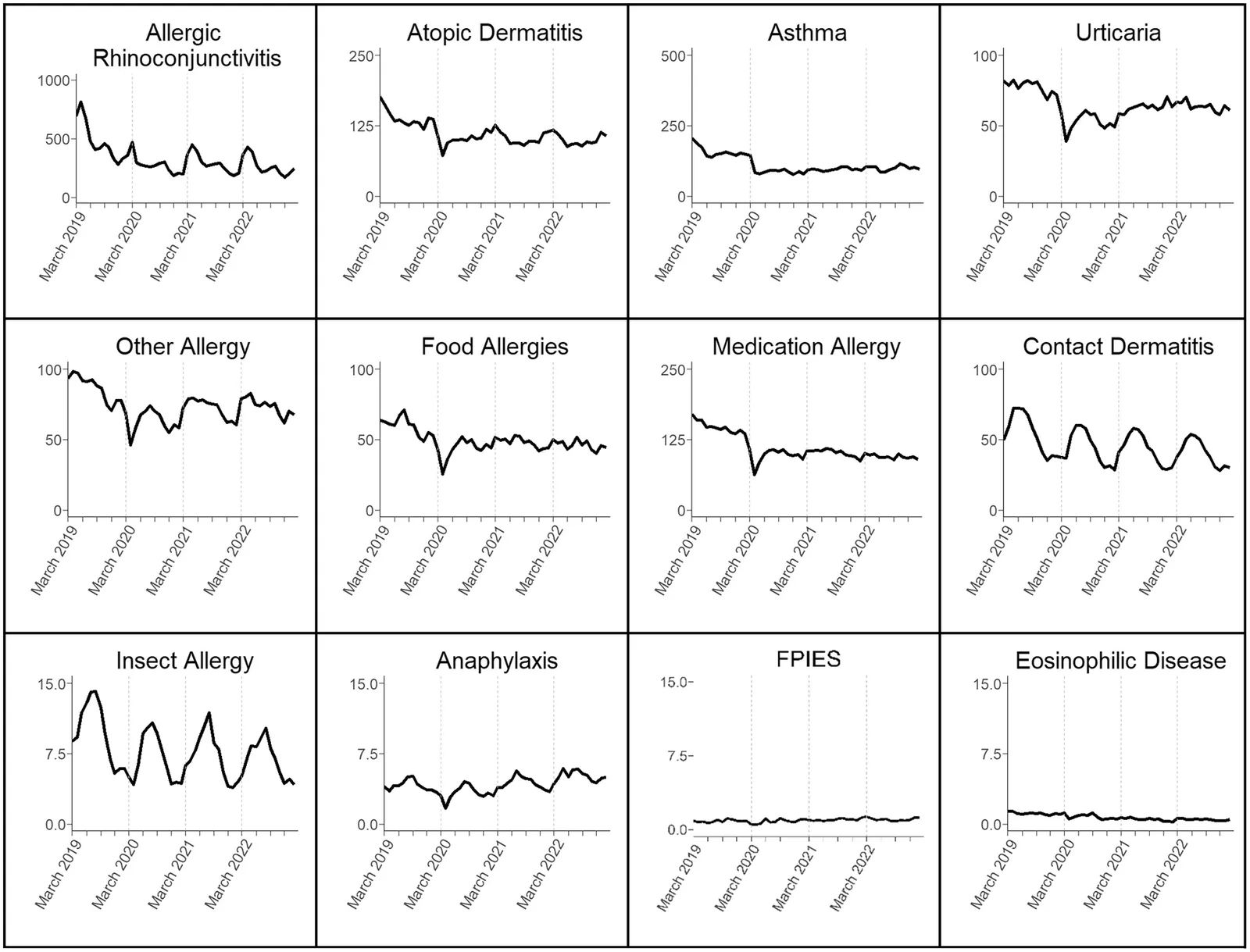
Incident rates by month for each of the 12 allergy diagnosis categories for the entire population. Rates are given as the number of incident cases per 100,000 people. All. rhinoconjunctivitis, allergic rhinoconjunctivitis; FPIES, food protein induced enterocolitis syndrome*.*

**Figure 2:**
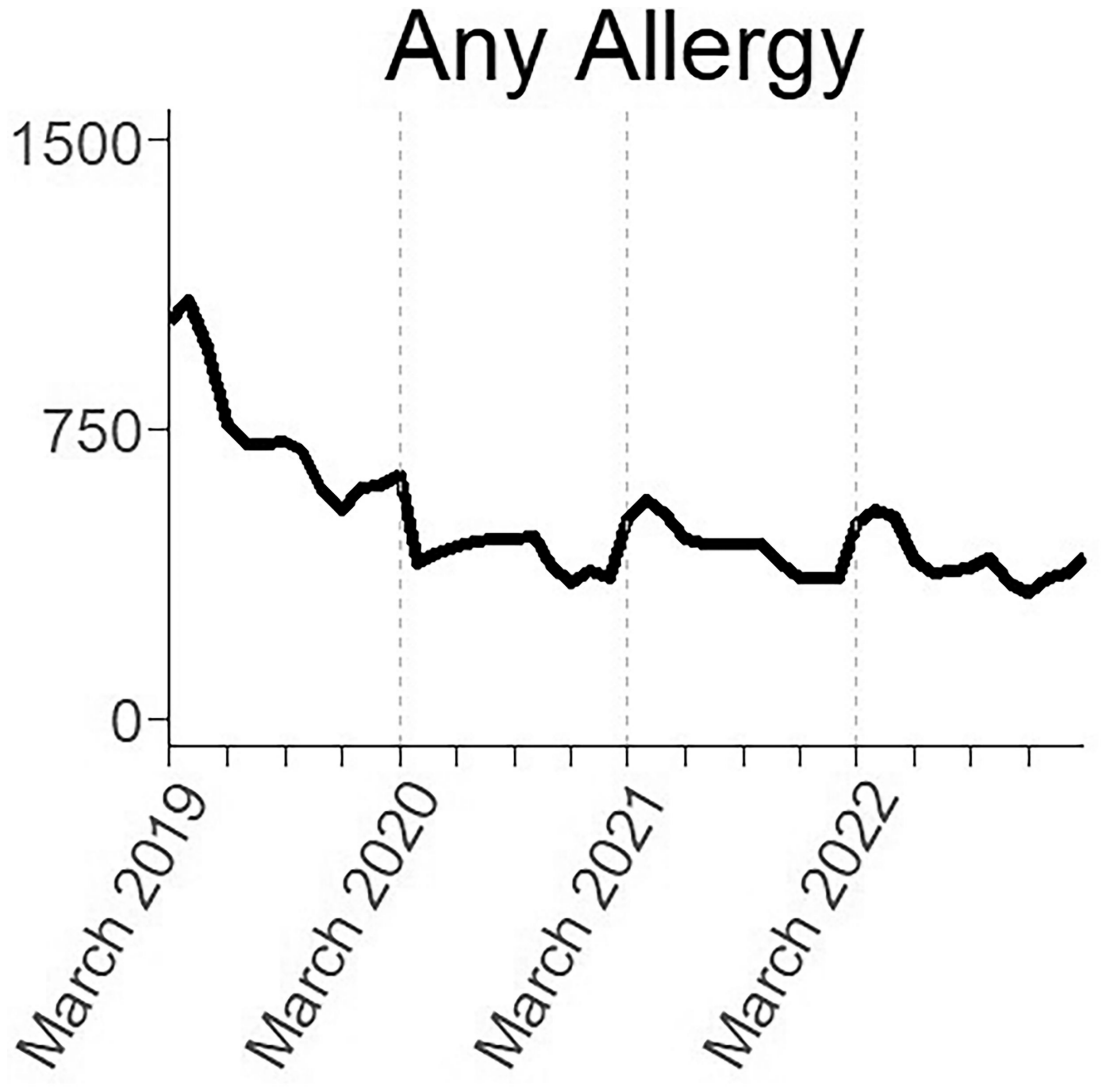
Incident rates by month for any of the 12 allergy diagnoses. Rates are given as the number of incident cases per 100,000 people.

Reductions in mean monthly incident rates from the pre-pandemic year to COVID-19 year 1 were statistically significant for all allergic diseases analyzed, with Bonferroni-corrected incident rate ratios ranging from 0.64 (0.62–0.66) for eosinophilic disease to 0.91 (0.85–0.97) for FPIES. The other ten allergic conditions studied also showed similar significant rate reductions (Table 3). During COVID-19 years 2 and 3, allergy diagnoses of all conditions, except for anaphylaxis and FPIES, remained lower compared with the pre-pandemic year (Table 3, Figure 1). Incident rates for development of any allergic disease were also significantly decreased in all three pandemic years, with incident rate ratios of 0.68 (0.68–0.68), 0.66 (0.66–0.66), and 0.61 (0.61–0.61) in pandemic years 1, 2, and 3, respectively (Table 3, Figure 2).

**Table 3:** Adjusted rate ratios of allergy diagnoses compared to pre-COVID year 2019, entire population (Bonferroni correction for 12 conditions applied to all conditions except any allergic condition).

Condition	COVID-19 year 1	COVID-19 year 2	COVID-19 year 3
Any allergic condition	0.68 (0.68–0.68)	0.66 (0.66–0.66)	0.61 (0.61–0.61)
All. rhinoconjunctivitis	0.70 (0.70–0.71)	0.69 (0.68–0.69)	0.65 (0.65–0.65)
Asthma	0.84 (0.84–0.85)	0.86 (0.86–0.86)	0.87 (0.87–0.88)
Medication allergy	0.76 (0.76–0.76)	0.76 (0.76–0.76)	0.77 (0.76–0.77)
Atopic dermatitis	0.86 (0.86–0.87)	0.93 (0.92–0.93)	0.92 (0.91–0.92)
Other allergy	0.86 (0.85–0.86)	0.90 (0.89–0.90)	0.92 (0.91–0.92)
Urticaria	0.85 (0.85–0.86)	0.89 (0.89–0.90)	0.88 (0.88–0.89)
Food allergies	0.77 (0.77–0.78)	0.79 (0.79–0.80)	0.76 (0.76–0.77)
Contact dermatitis	0.89 (0.89–0.90)	0.88 (0.87–0.88)	0.86 (0.85–0.86)
Insect allergy	0.78 (0.77–0.79)	0.80 (0.79–0.80)	0.76 (0.76–0.77)
Anaphylaxis	0.84 (0.83–0.85)	1.04 (1.03–1.06)	1.15 (1.14–1.17)
FPIES	0.91 (0.85–0.97)	1.10 (1.03–1.16)	1.28 (1.21–1.35)
Eosinophilic disease	0.64 (0.62–0.66)	0.36 (0.35–0.38)	0.39 (0.37–0.40)

All. rhinoconjunctivitis, allergic rhinoconjunctivitis; FPIES, food protein induced enterocolitis syndrome.

**Figure 3:**
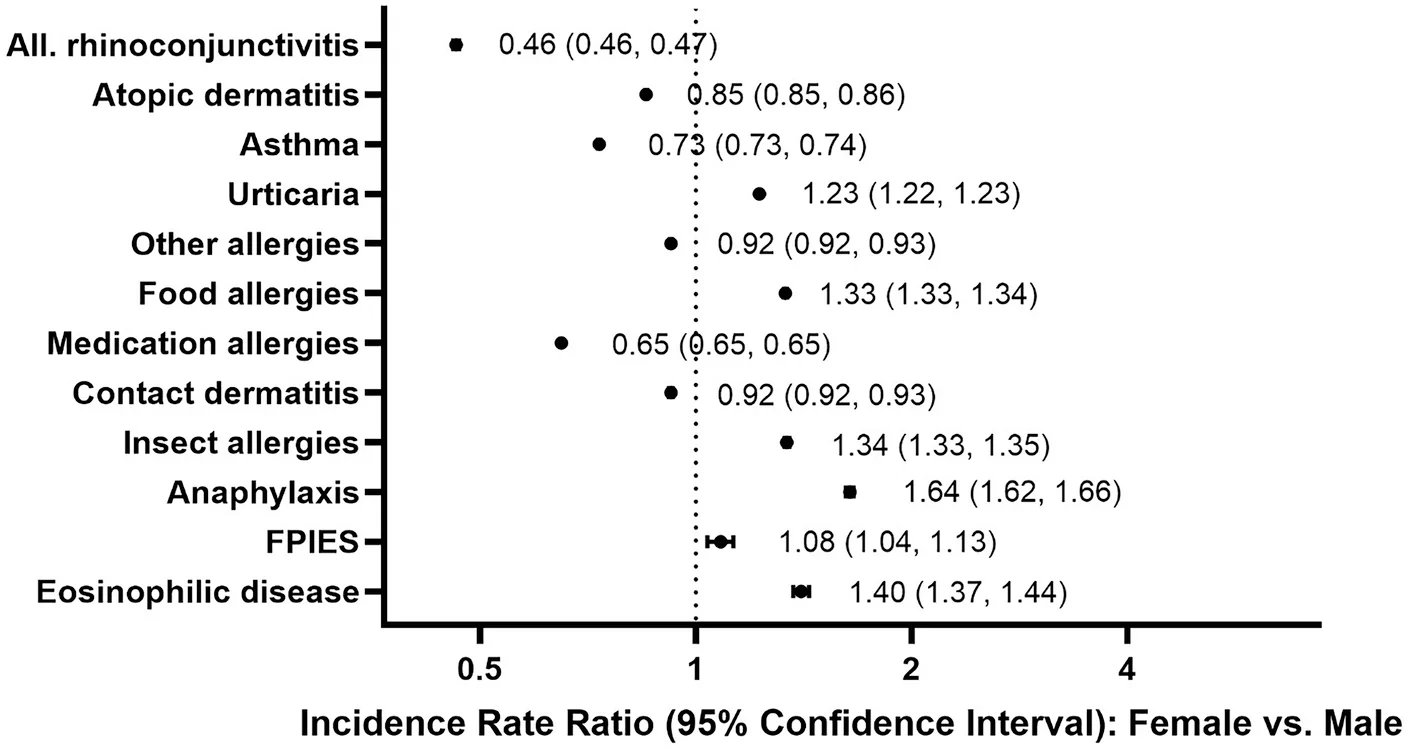
Sex differences in incident rates by allergy category. All. rhinoconjunctivitis, allergic rhinoconjunctivitis; FPIES, food protein induced enterocolitis syndrome.

Additional, *post-hoc* secondary analyses were conducted to evaluate if trends in frequencies of allergic diagnoses differed based on the immunological mechanism of allergic disease. Conditions were categorized as being primarily IgE-mediated (allergic rhinitis, food allergy, insect allergy, and anaphylaxis), primarily non-IgE mediated (contact dermatitis and FPIES), and mixed (asthma, urticaria, medication allergy, atopic dermatitis, eosinophilic diseases, and other allergy). As seen in Supplementary Table 3, adjusted rate ratios for all three categories were lower in each of the COVID years compared to 2019, though they were generally lowest for IgE-mediated conditions. With regards to demographic factors, incidence rates for allergic rhinoconjunctivitis, atopic dermatitis, asthma, other allergies, medication allergies, and contact dermatitis were all lower for females compared to males (Figure 3). In contrast, incidence rates for urticaria, food allergies, insect allergies, anaphylaxis, FPIES, and eosinophilic diseases were all higher for females (Figure 3). Of the four most commonly diagnosed allergies, individuals whose sponsor held the rank of Officer had the highest incidence rate for allergic rhinoconjunctivitis, and atopic dermatitis, while individuals whose sponsor held the rank of Junior Enlisted had the highest incidence rate for asthma and medication allergy (Supplementary Table 4). For all four of these allergies, individuals whose sponsor held the rank of Senior Enlisted had the lowest incidence rates (Supplementary Table 4).

**Figure 4:**
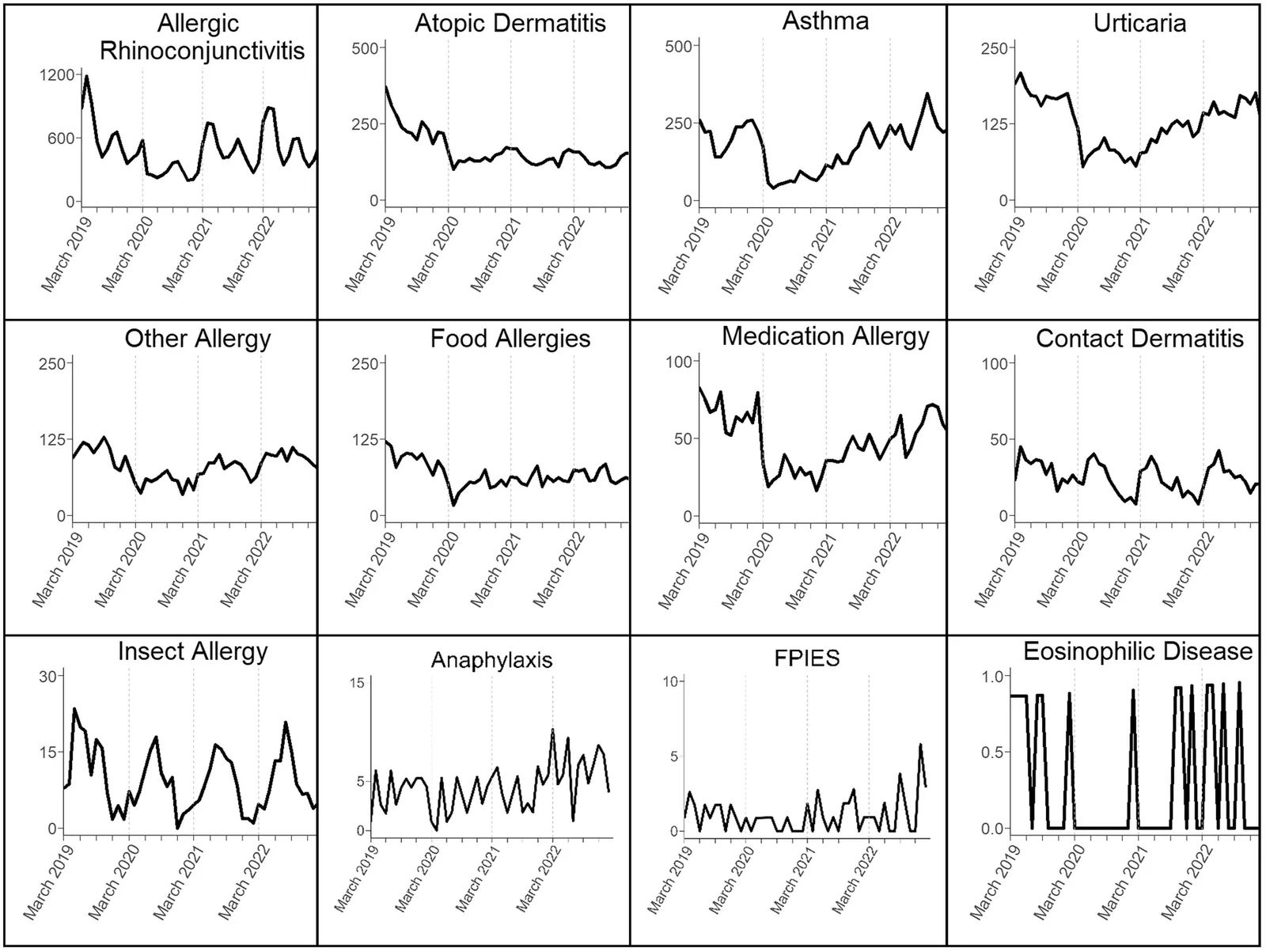
Incident rates by month for each of the 12 diagnoses for all 12 allergy categories for 3 year olds. Rates are given as the number of incident cases per 100,000 people. All. rhinoconjunctivitis, allergic rhinoconjunctivitis; FPIES, food protein induced enterocolitis syndrome.

**Figure 5:**
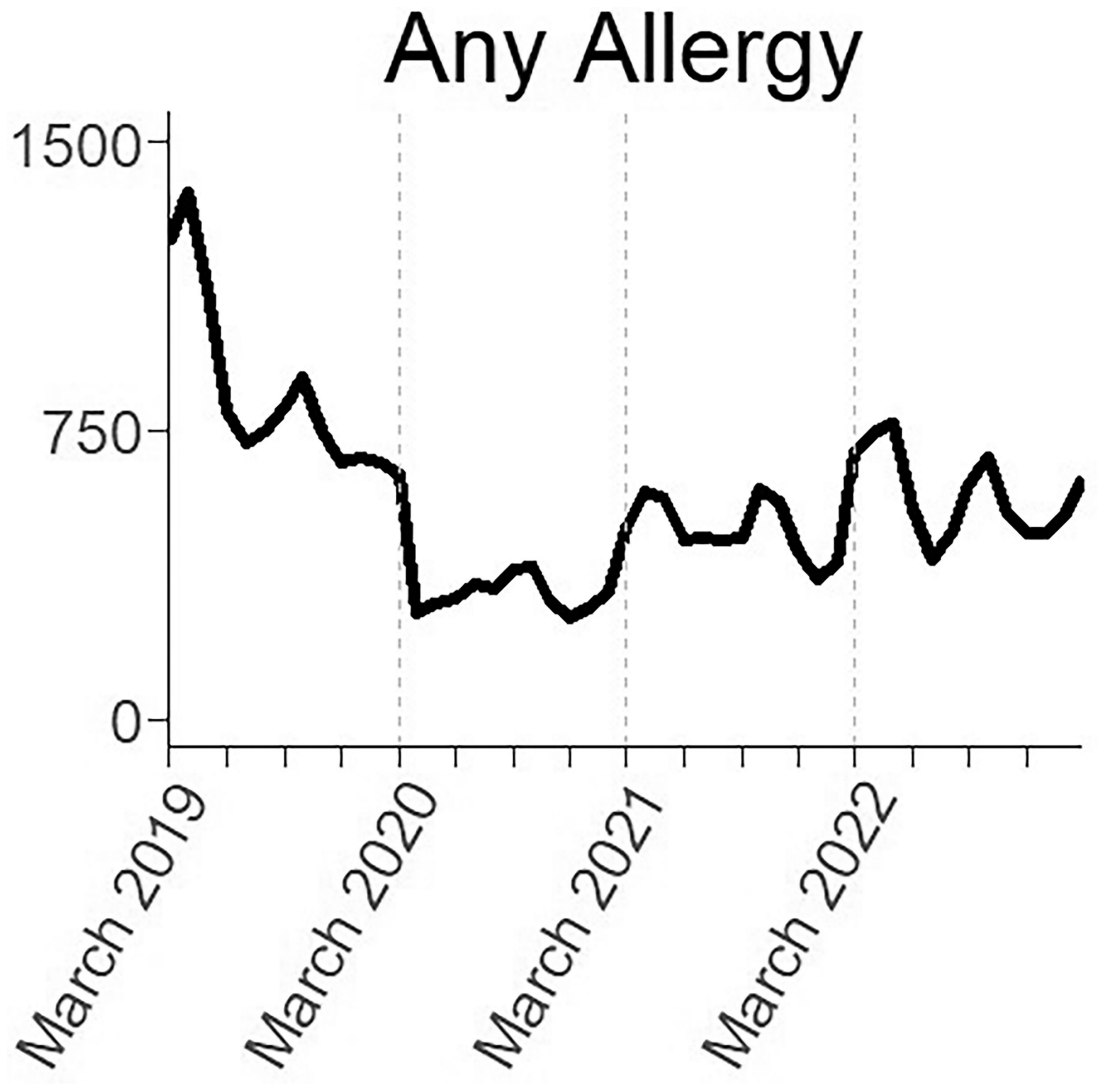
Incident rates by month for any of the 12 allergy diagnoses in 3-year-olds only. Rates are given as the number of incident cases per 100,000 people.

In the subanalysis of 3-year-olds, the most commonly diagnosed allergic diseases were allergic rhinoconjunctivitis, atopic dermatitis, asthma, and urticaria, with mean monthly incident rates per 100,000 of 616, 247, 213, and 172 during the pre-pandemic year, respectively (Table 4). During COVID-19 year 1, the mean monthly incident rates for these conditions decreased to 294, 137, 74, and 78 per 100,000 3-year-olds, respectively (Table 4, Figure 4). As was seen with the entire cohort, incident rates of all allergic diseases were significantly lower during COVID-19 year 1 compared to the pre-pandemic year, with adjusted rate ratios of 0.49 (CI 0.47–0.51) for allergic rhinoconjunctivitis, 0.61 (CI 0.57–0.64) for atopic dermatitis, 0.44 (CI 0.41–0.48) for asthma, and 0.50 (CI 0.47–0.54) for urticaria (Table 5). While mean monthly incident rates increased back to baseline by COVID-19 year 3 for asthma, anaphylaxis, and FPIES, they remained significantly lower during COVID-19 year 3 for the other allergic diagnoses as determined by adjusted rate ratios (Figure 4 and Table 5). As with the entire cohort incident rates for development of any allergic disease were significantly decreased in all three pandemic years (Table 5, Figure 5).

**Table 4:** Mean monthly diagnoses and monthly incident rates per 100,000 people of allergic conditions, 3 year olds.

Condition	Pre-COVID-19	COVID-19 year 1	COVID-19 year 2	COVID-19 year 3
Any allergic condition	1,002 (872.76)	391 (350.58)	536 (491.81)	612 (580.80)
All. rhinoconjunctivitis	708 (616.10)	327 (293.86)	532 (487.63)	578 (547.58)
Atopic dermatitis	283 (246.66)	152 (136.83)	150 (137.98)	140 (132.56)
Asthma	244 (212.97)	83 (74.16)	180 (165.35)	252 (239.16)
Urticaria	198 (172.35)	87 (77.95)	117 (107.90)	158 (150.29)
Other allergy	116 (101.24)	61 (54.42)	84 (77.46)	100 (95.07)
Food allergies	107 (93.26)	54 (48.87)	65 (59.22)	69 (65.28)
Medication allergy	78 (67.59)	30 (27.09)	45 (41.64)	60 (57.16)
Contact dermatitis	34 (29.84)	25 (22.32)	24 (21.82)	27 (25.93)
Insect allergy	13 (11.45)	10 (8.97)	9 (8.47)	10 (9.10)
Anaphylaxis	5 (4.08)	4 (3.27)	5 (4.14)	7 (6.41)
FPIES	2 (1.55)	1 (0.89)	2 (1.72)	3 (2.60)
Eosinophilic disease	1 (0.87)	1 (0.91)	1 (0.93)	1 (0.94)

All. rhinoconjunctivitis, allergic rhinoconjunctivitis; FPIES, food protein induced enterocolitis syndrome.

**Table 5:** Adjusted rate ratios of allergy diagnoses compared to pre-COVID year 2019, 3 year olds (Bonferroni correction for 12 conditions applied to all conditions except any allergic condition).

Condition	COVID-19 year 1	COVID-19 year 2	COVID-19 year 3
Any allergic condition	0.41 (0.40–0.42)	0.56 (0.55–0.58)	0.64 (0.63–0.66)
All. rhinoconjunctivitis	0.49 (0.47–0.51)	0.74 (0.71–0.77)	0.84 (0.81–0.87)
Atopic dermatitis	0.61 (0.57–0.64)	0.57 (0.53–0.60)	0.58 (0.55–0.62)
Asthma	0.44 (0.41–0.48)	0.72 (0.67–0.76)	0.98 (0.93–1.04)
Urticaria	0.50 (0.47–0.54)	0.64 (0.60–0.69)	0.84 (0.79–0.89)
Other allergy	0.60 (0.55–0.65)	0.66 (0.62–0.72)	0.82 (0.76–0.88)
Food allergies	0.61 (0.56–0.66)	0.67 (0.62–0.72)	0.78 (0.72–0.84)
Medication allergy	0.55 (0.50–0.60)	0.70 (0.65–0.77)	0.83 (0.77–0.90)
Contact dermatitis	0.67 (0.60–0.74)	0.63 (0.57–0.71)	0.69 (0.62–0.77)
Insect allergy	0.65 (0.56–0.75)	0.57 (0.49–0.67)	0.69 (0.59–0.80)
Anaphylaxis	0.64 (0.50–0.80)	0.78 (0.62–0.97)	1.23 (1.01–1.50)
FPIES	0.30 (0.18–0.52)	0.75 (0.51–1.12)	1.03 (0.72–1.49)
Eosinophilic disease	0.10 (0.03–0.36)	0.32 (0.15–0.69)	0.44 (0.22–0.89)

All. rhinoconjunctivitis, allergic rhinoconjunctivitis; FPIES, food protein induced enterocolitis syndrome.

## Discussion

The goal of this study was to determine changes in yearly incidence rates of allergic diseases over the first 3 years of the COVID-19 pandemic. We found that the incidence rates of all allergy diagnoses evaluated (allergic rhinoconjunctivitis, asthma, medication allergy, atopic dermatitis, other allergy, urticaria, food allergy, contact dermatitis, insect allergy, anaphylaxis, eosinophilic disease, and FPIES) decreased significantly during year 1 of the COVID-19 pandemic, and that the incidence of all allergy diagnoses except anaphylaxis and FPIES remained lower during COVID-19 years 2 and 3 compared to the pre-pandemic year. Although our study cannot determine a causal link for this decrease, we discuss below some potential reasons for the decrease in diagnoses.

The most straightforward explanation for this finding would be that allergic diagnoses decreased during the pandemic due to decreased healthcare utilization. Indeed, a decline in encounters with healthcare providers occurred during the first year of the COVID-19 pandemic as billed ambulatory visits per 1,000 person years, for active duty members in the MHS, decreased from 15,887 in 2019 to 14,719 in 2020 (7.5% decrease from 2019 to 2020) (7). That stated, this decrease in healthcare encounters was substantially less than the decrease in allergy diagnoses that occurred during the 1st pandemic year, which had an incident rate ratio for all allergy diagnoses of 0.68 with respect to the pre-pandemic year. It would be expected that if decreased healthcare contact was the cause of reduced allergy diagnoses, a “rebound” period of increased allergy diagnoses would be seen after this effect subsided. However, this was not seen during the study period which continued until February 2023. Moreover, total clinical visits in the MHS rebounded to pre-pandemic levels by 2021 and 2022 (7), time periods when incidence rates of allergy diagnoses, except for anaphylaxis and FPIES, remained significantly lower than in the pre-pandemic year. Consequently, while reduced healthcare utilization may have initially contributed to some of the decline observed in incident rates of allergy, it appears likely that the decreased incidence rates of allergy diagnoses we observed in the MHS during the first 3 years of the COVID-19 pandemic reflect a true decrease in the incidence of allergic diseases.

The mechanisms by which the COVID-19 pandemic may have decreased incidence rates of allergy diagnoses are not entirely clear. One possible explanation for decreasing allergy diagnoses may be lower population exposure to common seasonal viruses. It is well recognized that viral illness can be a cause of asthma and other atopic exacerbations (4, 8). During the COVID-19 pandemic, due to stay-at-home orders, masking, and increased isolation, the incidence of influenza and respiratory syncytial virus decreased dramatically, with both estimated to be 70%–90% lower than usual during the first year of the COVID-19 pandemic (9). Thus, the reduction in exposure to respiratory virus infections may have been a key factor driving the observed decline in atopic conditions during the first 3 years of the COVID-19 pandemic. Additionally, it is possible that alterations in behavior led to changes in exposures to common allergens such as reduced exposure to outdoor allergens, allergens in the workplace, or allergens from reduced use of contact allergens such as cosmetics that may have been used less frequently during the pandemic.

Notably, we did not observe any increase in allergy diagnoses. At the onset of the COVID-19 pandemic, there was some concern, largely based on the hygiene hypothesis, that reduced exposures to infectious agents could potentially result in reduced microbiome diversity with subsequent immune dysregulation and increased rates of inflammatory diseases such as allergy (1). Not only did we not observe any increases in allergies in the entire cohort studied, we also did not observe any increases in the sub-analysis of 3-year olds, who one would expect to be especially susceptible to changes in microbial exposures (10). That stated, our study's findings do not necessarily invalidate the hygiene hypothesis as baseline microbial exposures may have already been too low to induce significant immune regulation even before the pandemic began.

To date, a few other studies have reported on allergy diagnoses during the COVID-19 pandemic, and the findings have been mixed. The Cow's Milk Early Exposure Trial (COMEET) investigated 2,252 infants born in Israel from April 2018–May 2021 and analyzed the development of atopic dermatitis, airway hypersensitivity and food allergy in the first 12 months of life (11). While COMEET found no significant difference in the development of atopic diseases between infants born before and during the COVID-19 pandemic, it did observe a higher incidence of airway hypersensitivity in children born after the pandemic. More in line with our study are findings from the CORAL birth cohort, which studied 344 infants born in Ireland during the first weeks of the COVID-19 pandemic and then followed them for 24 months (12). The CORAL study observed a reduced incidence of milk, peanut and egg allergies, but a higher rate of atopic dermatitis in the children in their cohort compared with a national Irish dataset from before the COVID-19 pandemic.

With respect to asthma, several studies have noted a decrease in exacerbations and emergency department visits compared to baseline during the COVID-19 pandemic. During the first 4 months after the stay-at-home order in 2020, the number of daily emergency department visits for asthma at the Children's Hospital of Philadelphia decreased from 24.3 to 5.8 (76% reduction) (13), and at Boston Children's Hospital an 82% decrease in Emergency Department visits for asthma was observed between March and May 2020 (4, 14). The Centers for Disease Control and Prevention also observed a nationwide decline in asthma emergency department visits, hospitalizations and deaths in 2020 compared to prior years (15).

Reductions in asthma diagnoses observed in our study and others (4, 13, 14) were more likely due to behavioral changes and reduced population exposures to all common respiratory viruses, occupational allergens, and contact allergens than to a direct effect of SARS-CoV-2 infection, as several studies have observed that SARS-CoV-2 infection is a risk factor for allergic asthma. Kim et al., in a retrospective cohort study, noted an increased risk of new-onset asthma after COVID-19 infection (16). Similarly, a retrospective cohort study analyzing nationwide claims-based cohorts in South Korea (*n* = 836,164), Japan (*n* = 2,541,021) and the UK Biobank cohort (*n* = 325,843) found that patients with a prior diagnosis of COVID-19 were at increased risk of developing asthma and allergic rhinitis, but not at increased risk of developing atopic dermatitis compared to those without a diagnosis of COVID-19 (17). Interestingly, COVID-19 vaccination of at least two doses negated this increased risk, suggesting that severity of COVID-19 infection may impact future risk of asthma development.

This study has several limitations. The retrospective design prevents us from determining causation and limits our ability to directly account for potential confounders such as healthcare utilization. Additionally, reliance on ICD-10 diagnoses may result in misclassification due to coding errors and may not capture when more than one disease is contributing to a clinical presentation. Another limitation is the use of a relatively short 1-year period (2018–2019) to identify prior allergy diagnoses. Because we only analyzed data for 1 year prior to the pre-pandemic year, we were not able to conduct an interrupted time series analysis, which would have enabled assessment of whether the decreasing frequencies observed during the pandemic were a new change or a continuation of a pre-existing trend. Furthermore, the 1 year period was likely too short to diagnose all previously documented cases of allergic disease, and thus some diagnoses identified as incident allergy cases may have actually been recurrent ones. That stated, misclassifications of prevalent or recurrent diagnoses as incident diagnoses are not anticipated to differ between the pre-pandemic year and post-pandemic years, and thus would not be expected to affect the trends in total incident diagnoses per year. Lack of data regarding adherence to COVID-19 mitigation measures and other environmental exposures limited our ability to determine how much these factors could have accounted for changes in allergy diagnoses. Finally, even with a sub-analysis based on how IgE impacts each diagnosis, the biological heterogeneity of the atopic conditions studied makes it challenging to draw conclusions about the biological mechanisms that resulted in lower allergy diagnoses during the pandemic.

While the study has several limitations, it also has a number of strengths. First, the analyzed population was large (>7 million people) and geographically, demographically, and socioeconomically diverse. Second, as individuals in the Military Health System, everyone in the population studied had access to universal healthcare. Thus, there were limited financial barriers to seeking medical care and consequently patients were likely able to visit a healthcare provider, either in person or via telehealth, if they developed allergic symptoms. Finally, the study controlled for numerous potential confounders, including age, sex, geographic location, and military rank (as a surrogate for income and education level).

Given the available access to healthcare within the MHS, and that total MHS clinic visits returned to pre-pandemic levels in years 2 and 3 of the COVID-19 pandemic, we believe the results of this study convincingly show that the incidence of allergic diseases significantly decreased over the first 3 years of the COVID-19 pandemic. To confirm these findings, additional retrospective studies in other populations with universal healthcare access are recommended, as these settings minimize financial barriers to diagnosis and ensure a more complete capture of true disease incidence. If our findings are reproduced, then future work should aim to identify the specific behavioral or environmental factors altered by the COVID-19 pandemic that may have contributed to the observed declines in allergic disease rates.

## Data Availability

The raw data supporting the conclusions of this article will be made available by the authors, without undue reservation.

## Author disclaimer

The views expressed in this presentation are the sole responsibility of the presenter and do not necessarily reflect the views, opinions, or policies of the Uniformed Services University of the Health Sciences, the Department of War, the Department of the Navy, Army, Air Force, Defense Health Agency, the United States Government, or the Henry M. Jackson Foundation for the Advancement of Military Medicine, Inc. (HJF). Several of the authors are U.S. Government employees. This work was prepared as part of their official duties. Title 17 US.C. § 105 provides that “Copyright protection under this title is not available for any work of the United States Government”. Title 17 US.C. § 101 defines a U.S. Government work as a work prepared by a military service member or employee of the U.S. Government as part of that person's official duties. Mention of trade names, commercial products, or organizations does not imply endorsement by the U.S. Government. The study protocol was approved by the USU Institutional Review Board in compliance with all applicable federal regulations governing the protection of human subjects.
