# Supplementary material for: Incidence rates of allergy diagnoses in the U.S. military health system after the COVID-19 pandemic
Source: Front Allergy. 2026 Aug 21;7:1884771. doi: 10.3389/falgy.2026.1884771 (PMC13541455; doi:10.3389/falgy.2026.1884771)
Supplement: Supplementary file 1 [file Table1.docx]

**Supplementary Table 1: ICD-10 Codes and Categories used in Study**

| **ICD-10 Code** | **Study Category** | **ICD-10 Associated Name** |
| --- | --- | --- |
| H1045 | Allergic rhinoconjunctivitis | Other chronic allergic conjunctivitis |
| J301 | Allergic rhinoconjunctivitis | Allergic rhinitis due to pollen |
| J302 | Allergic rhinoconjunctivitis | Other seasonal allergic rhinitis |
| J3081 | Allergic rhinoconjunctivitis | Allergic rhinitis due to animal (cat) (dog) hair and dander |
| J3089 | Allergic rhinoconjunctivitis | Other allergic rhinitis |
| J309 | Allergic rhinoconjunctivitis | Allergic rhinitis, unspecified |
| J301 | Allergic rhinoconjunctivitis | Allergic rhinitis due to pollen |
| J302 | Allergic rhinoconjunctivitis | Other seasonal allergic rhinitis |
| J3081 | Allergic rhinoconjunctivitis | Allergic rhinitis due to animal (cat) (dog) hair and dander |
| J3089 | Allergic rhinoconjunctivitis | Other allergic rhinitis |
| J309 | Allergic rhinoconjunctivitis | Allergic rhinitis, unspecified |
| J4520 | Asthma | Mild intermittent asthma, uncomplicated |
| J4521 | Asthma | Mild intermittent asthma with (acute) exacerbation |
| J4522 | Asthma | Mild intermittent asthma with status asthmaticus |
| J4530 | Asthma | Mild persistent asthma, uncomplicated |
| J4531 | Asthma | Mild persistent asthma with (acute) exacerbation |
| J4532 | Asthma | Mild persistent asthma with status asthmaticus |
| J4540 | Asthma | Moderate persistent asthma, uncomplicated |
| J4541 | Asthma | Moderate persistent asthma with (acute) exacerbation |
| J4542 | Asthma | Moderate persistent asthma with status asthmaticus |
| J4550 | Asthma | Severe persistent asthma, uncomplicated |
| J4551 | Asthma | Severe persistent asthma with (acute) exacerbation |
| J4552 | Asthma | Severe persistent asthma with status asthmaticus |
| J45901 | Asthma | Unspecified asthma with (acute) exacerbation |
| J45902 | Asthma | Unspecified asthma with status asthmaticus |
| J45909 | Asthma | Unspecified asthma, uncomplicated |
| J45990 | Asthma | Exercise induced bronchospasm |
| J45991 | Asthma | Cough variant asthma |
| J45998 | Asthma | Other asthma |
| Z880 | Medication allergy | Allergy status to penicillin |
| Z881 | Medication allergy | Allergy status to other antibiotic agents status |
| Z882 | Medication allergy | Allergy status to sulfonamides status |
| Z883 | Medication allergy | Allergy status to other anti-infective agents status |
| Z884 | Medication allergy | Allergy status to anesthetic agent status |
| Z885 | Medication allergy | Allergy status to narcotic agent status |
| Z886 | Medication allergy | Allergy status to analgesic agent status |
| Z887 | Medication allergy | Allergy status to serum and vaccine status |
| Z888 | Medication allergy | Allergy status to oth drug/meds/biol subst status |
| Z889 | Medication allergy | Allergy status to unsp drug/meds/biol subst status |
| H01111 | Atopic dermatitis | Allergic dermatitis of right upper eyelid |
| H01112 | Atopic dermatitis | Allergic dermatitis of right lower eyelid |
| H01113 | Atopic dermatitis | Allergic dermatitis of right eye, unspecified eyelid |
| H01114 | Atopic dermatitis | Allergic dermatitis of left upper eyelid |
| H01115 | Atopic dermatitis | Allergic dermatitis of left lower eyelid |
| H01116 | Atopic dermatitis | Allergic dermatitis of left eye, unspecified eyelid |
| H01119 | Atopic dermatitis | Allergic dermatitis of unspecified eye, unspecified eyelid |
| H01131 | Atopic dermatitis | Eczematous dermatitis of right upper eyelid |
| H01132 | Atopic dermatitis | Eczematous dermatitis of right lower eyelid |
| H01133 | Atopic dermatitis | Eczematous dermatitis of right eye, unspecified eyelid |
| H01134 | Atopic dermatitis | Eczematous dermatitis of left upper eyelid |
| H01135 | Atopic dermatitis | Eczematous dermatitis of left lower eyelid |
| H01136 | Atopic dermatitis | Eczematous dermatitis of left eye, unspecified eyelid |
| H01139 | Atopic dermatitis | Eczematous dermatitis of unspecified eye, unspecified eyelid |
| L200 | Atopic dermatitis | Besniers prurigo |
| L2081 | Atopic dermatitis | Atopic neurodermatitis |
| L2082 | Atopic dermatitis | Flexural eczema |
| L2083 | Atopic dermatitis | Infantile (acute) (chronic) eczema |
| L2084 | Atopic dermatitis | Intrinsic (allergic) eczema |
| L2089 | Atopic dermatitis | Other atopic dermatitis |
| L209 | Atopic dermatitis | Atopic dermatitis, unspecified |
| L300 | Atopic dermatitis | Nummular dermatitis |
| L300 | Atopic dermatitis | Nummular dermatitis |
| L301 | Atopic dermatitis | Dyshidrosis [pompholyx] |
| L2081 | Atopic dermatitis | Atopic neurodermatitis |
| L2082 | Atopic dermatitis | Flexural eczema |
| L2083 | Atopic dermatitis | Infantile (acute) (chronic) eczema |
| L2084 | Atopic dermatitis | Intrinsic (allergic) eczema |
| L2089 | Atopic dermatitis | Other atopic dermatitis |
| L209 | Atopic dermatitis | Atopic dermatitis, unspecified |
| L272 | Atopic dermatitis | Dermatitis due to ingested food |
| D690 | Other allergy | Allergic purpura |
| K522 | Other allergy | Allergic and dietetic gastroenteritis and colitis |
| T783XXA | Other allergy | Angioneurotic edema, initial encounter |
| T7840XA | Other allergy | Allergy, unspecified, initial encounter |
| T7849XA | Other allergy | Other allergy, initial encounter |
| T783XXD | Other allergy | Angioneurotic edema, subsequent encounter |
| T7840XD | Other allergy | Allergy, unspecified, subsequent encounter |
| T7849XD | Other allergy | Other allergy, subsequent encounter |
| K522 | Other allergy | Allergic and dietetic gastroenteritis and colitis |
| K5229 | Other allergy | Other allergic and dietetic gastroenteritis and colitis |
| Z91041 | Other allergy | Radiographic dye allergy status |
| Z91048 | Other allergy | Other nonmedicinal substance allergy status |
| Z9109 | Other allergy | Oth allergy status, oth than to drugs and biolg substances |
| L500 | Urticaria | Allergic urticaria |
| L501 | Urticaria | Idiopathic urticaria |
| L502 | Urticaria | Urticaria due to cold and heat |
| L505 | Urticaria | Cholinergic urticaria |
| L506 | Urticaria | Contact urticaria |
| L508 | Urticaria | Other urticaria |
| L509 | Urticaria | Urticaria, unspecified |
| L500 | Urticaria | Allergic urticaria |
| L501 | Urticaria | Idiopathic urticaria |
| L505 | Urticaria | Cholinergic urticaria |
| L508 | Urticaria | Other urticaria |
| L509 | Urticaria | Urticaria, unspecified |
| T7808XA | Food allergy | Anaphylactic reaction due to eggs, initial encounter |
| T7808XD | Food allergy | Anaphylactic reaction due to eggs, subsequent encounter |
| Z91012 | Food allergy | Allergy to eggs |
| T7803XA | Food allergy | Anaphylactic reaction due to other fish, initial encounter |
| T7803XD | Food allergy | Anaphylactic reaction due to other fish, subs encntr |
| T7806XA | Food allergy | Anaphylactic reaction due to food additives, init encntr |
| T7806XD | Food allergy | Anaphylactic reaction due to food additives, subs encntr |
| Z9102 | Food allergy | Food additives allergy status |
| T7804XA | Food allergy | Anaphylactic reaction due to fruits and vegetables, init |
| T7804XD | Food allergy | Anaphylactic reaction due to fruits and vegetables, subs |
| T7807XA | Food allergy | Anaphylactic reaction due to milk and dairy products, init |
| T7807XD | Food allergy | Anaphylactic reaction due to milk and dairy products, subs |
| Z91011 | Food allergy | Allergy to milk products |
| T7809XA | Food allergy | Anaphylactic reaction due to oth food products, init encntr |
| T7809XD | Food allergy | Anaphylactic reaction due to oth food products, subs encntr |
| Z91018 | Food allergy | Allergy to other foods |
| T7801XA | Food allergy | Anaphylactic reaction due to peanuts, initial encounter |
| T7801XD | Food allergy | Anaphylactic reaction due to peanuts, subsequent encounter |
| Z91010 | Food allergy | Allergy to peanuts |
| Z91013 | Food allergy | Allergy to seafood |
| T7802XA | Food allergy | Anaphylactic reaction due to shellfish (crustaceans), init |
| T7802XD | Food allergy | Anaphylactic reaction due to shellfish (crustaceans), subs |
| T7805XA | Food allergy | Anaphylactic reaction due to tree nuts and seeds, init |
| T7805XD | Food allergy | Anaphylactic reaction due to tree nuts and seeds, subs |
| J305 | Food allergy | Allergic rhinitis due to food |
| L272 | Food allergy | Dermatitis due to ingested food |
| T7800XA | Food allergy | Anaphylactic reaction due to unspecified food, init encntr |
| T7800XD | Food allergy | Anaphylactic reaction due to unspecified food, subs encntr |
| J305 | Food allergy | Allergic rhinitis due to food |
| L230 | Contact dermatitis | Allergic contact dermatitis due to metals |
| L231 | Contact dermatitis | Allergic contact dermatitis due to adhesives |
| L232 | Contact dermatitis | Allergic contact dermatitis due to cosmetics |
| L233 | Contact dermatitis | Allergic contact dermatitis due to drugs in contact with skin |
| L234 | Contact dermatitis | Allergic contact dermatitis due to dyes |
| L235 | Contact dermatitis | Allergic contact dermatitis due to other chemical products |
| L236 | Contact dermatitis | Allergic contact dermatitis due to food in contact with the skin |
| L237 | Contact dermatitis | Allergic contact dermatitis due to plants, except food |
| L2381 | Contact dermatitis | Allergic contact dermatitis due to animal (cat) (dog) dander |
| L2389 | Contact dermatitis | Allergic contact dermatitis due to other agents |
| L239 | Contact dermatitis | Allergic contact dermatitis, unspecified cause |
| L230 | Contact dermatitis | Allergic contact dermatitis due to metals |
| L231 | Contact dermatitis | Allergic contact dermatitis due to adhesives |
| L232 | Contact dermatitis | Allergic contact dermatitis due to cosmetics |
| L233 | Contact dermatitis | Allergic contact dermatitis due to drugs in contact with skin |
| L234 | Contact dermatitis | Allergic contact dermatitis due to dyes |
| L235 | Contact dermatitis | Allergic contact dermatitis due to other chemical products |
| L236 | Contact dermatitis | Allergic contact dermatitis due to food in contact with the skin |
| L237 | Contact dermatitis | Allergic contact dermatitis due to plants, except food |
| L2381 | Contact dermatitis | Allergic contact dermatitis due to animal (cat) (dog) dander |
| L2389 | Contact dermatitis | Allergic contact dermatitis due to other agents |
| L239 | Contact dermatitis | Allergic contact dermatitis, unspecified cause |
| Z91040 | Contact dermatitis | Latex allergy status |
| Z91030 | Insect allergy | Bee allergy status |
| Z91038 | Insect allergy | Other insect allergy status |
| T782XXA | Anaphylaxis | Anaphylactic shock, unspecified, initial encounter |
| T782XXD | Anaphylaxis | Anaphylactic shock, unspecified, subsequent encounter |
| K5221 | Food protein-induced enterocolitis syndrome | Food protein-induced enterocolitis syndrome |
| K5222 | Food protein-induced enterocolitis syndrome | Food protein-induced enteropathy |
| K5281 | Eosinophilic disease | Eosinophilic gastritis or gastroenteritis |
| K5282 | Eosinophilic disease | Eosinophilic colitis |
| J82 | Eosinophilic disease | Pulmonary eosinophilia, not elsewhere classified |
| L983 | Eosinophilic disease | Eosinophilic cellulitis [Wells] |

**Supplementary Table 2: Mean monthly median age of diagnosis for each of the 12 allergy categories median (IQR)**

| Condition | Pre-COVID-19 | COVID-19 Yr. 1 | COVID-19 Yr. 2 | COVID-19 Yr. 3 |
| --- | --- | --- | --- | --- |
| Any Allergic Condition | 27 (12, 46) | 28 (14, 45) | 25 (11, 41) | 24 (9, 40) |
| All. Rhinoconjunctivitis | 28 (12, 46) | 30 (15, 47) | 26 (10, 43) | 24 (9, 41) |
| Atopic Dermatitis | 15 (2, 37) | 15 (1, 37) | 16 (1, 38) | 17 (1, 38) |
| Asthma | 26 (12, 47) | 30 (18, 49) | 26 (12, 45) | 24 (8, 43) |
| Urticaria | 21 (6, 37) | 23 (9, 39) | 23 (8, 38) | 21 (6, 36) |
| Other Allergy | 30 (14, 49) | 31 (17, 49) | 30 (15, 47) | 29 (14, 46) |
| Food Allergies | 19 (6, 37) | 20 (4, 37) | 20 (4, 37) | 20 (3, 37) |
| Medication Allergy | 39 (25, 55) | 39 (25, 55) | 38 (24, 54) | 37 (23, 54) |
| Contact Dermatitis | 35 (20, 52) | 35 (21, 52) | 35 (21, 51) | 35 (21, 51) |
| Insect Allergy | 32 (17, 50) | 33 (17, 50) | 33 (17, 50) | 34 (18, 51) |
| Anaphylaxis | 28 (16, 44) | 28 (16, 43) | 28 (15, 43) | 26 (15, 41) |
| FPIES | 0 (0, 1) | 0 (0, 1) | 0 (0, 1) | 0 (0, 1) |
| Eosinophilic Disease | 39 (18, 56) | 34 (15, 53) | 24 (4, 42) | 31 (15, 46) |

Abbreviations: IQR: interquartile (25% and 75%); FPIES: food protein induced enterocolitis syndrome; All. Rhinoconjunctivitis: allergic rhinoconjunctivitis

**Supplementary Table 3: Adjusted rate ratios of allergy diagnoses compared to pre-COVID year 2019, entire population. (Bonferroni correction for 3 conditions applied to all conditions.)**

| **Condition** | **COVID-19 yr. 1** | **COVID-19 yr. 2** | **COVID-19 yr. 3** |
| --- | --- | --- | --- |
| **IgE-mediated allergic diseases** | 0.65 (0.64-0.65) | 0.62 (0.62-0.62) | 0.57 (0.56-0.57) |
| **Non-IgE-mediated conditions** | 0.84 (0.83-0.85) | 0.74 (0.73-0.75) | 0.68 (0.67-0.69) |
| **Mixed** | 0.69 (0.69-0.70) | 0.68 (0.67-0.68) | 0.66 (0.66-0.66) |

**Supplementary Table 4: Covariate incident rate ratios by allergy category.**

|  | | **Any allergic condition** | **All. rhinoconjunctivitis** | **Atopic dermatitis** | **Asthma** | **Urticaria** | **Other allergies** | **Food allergies** | **Medication allergies** | **Contact dermatitis** | **Insect allergies** | **Anaphylaxis** | **FPIES** | **Eosinophilic disease** |
| --- | --- | --- | --- | --- | --- | --- | --- | --- | --- | --- | --- | --- | --- | --- |
| **Active Duty** | Yes vs. No | 0.20 (0.20, 0.20) | 0.20 (0.20, 0.21) | 0.15 (0.15, 0.15) | 0.10 (0.10, 0.10) | 0.18 (0.18, 0.18) | 0.14 (0.13, 0.14) | 0.13 (0.12, 0.13) | 0.13 (0.13, 0.13) | 0.13 (0.12, 0.13) | 0.13 (0.13, 0.14) | 0.15 (0.14, 0.15) | 0.07 (0.06, 0.10) | 0.13 (0.12, 0.14) |
| **Age** | 1-5 vs. <1 year | 0.39 (0.39, 0.40) | 1.50 (1.47, 1.53) | 0.24 (0.24, 0.25) | 1.35 (1.31, 1.39) | 0.88 (0.86, 0.90) | 0.75 (0.73, 0.77) | 0.39 (0.38, 0.40) | 1.26 (1.22, 1.31) | 0.95 (0.91, 0.98) | 5.74 (5.02, 6.55) | 0.89 (0.83, 0.96) | 0.09 (0.08, 0.09) | 0.14 (0.13, 0.16) |
|  | 6-11 vs. <1 year | 0.34 (0.34, 0.34) | 1.43 (1.40, 1.45) | 0.22 (0.22, 0.23) | 1.58 (1.53, 1.62) | 0.86 (0.84, 0.88) | 0.94 (0.92, 0.96) | 0.43 (0.42, 0.44) | 1.76 (1.70, 1.82) | 1.54 (1.49, 1.60) | 8.43 (7.38, 9.62) | 1.28 (1.19, 1.38) | 0.02 (0.02, 0.03) | 0.20 (0.18, 0.22) |
|  | 12-17 vs. <1 year | 0.32 (0.32, 0.33) | 1.44 (1.41, 1.46) | 0.27 (0.26, 0.27) | 1.98 (1.93, 2.03) | 1.01 (0.99, 1.03) | 1.23 (1.20, 1.26) | 0.56 (0.55, 0.57) | 2.55 (2.46, 2.64) | 2.01 (1.93, 2.08) | 7.05 (6.18, 8.06) | 1.76 (1.63, 1.89) | 0.01 (0.01, 0.01) | 0.35 (0.32, 0.39) |
|  | 18-24 vs. <1 year | 0.70 (0.69, 0.70) | 2.65 (2.60, 2.70) | 0.51 (0.50, 0.51) | 4.09 (3.98, 4.19) | 2.08 (2.03, 2.12) | 2.62 (2.56, 2.68) | 1.10 (1.08, 1.12) | 6.59 (6.38, 6.82) | 4.98 (4.80, 5.15) | 17.06 (14.95, 19.46) | 3.59 (3.34, 3.85) | 0.02 (0.01, 0.02) | 0.50 (0.46, 0.55) |
|  | 25-50 vs. <1 year | 1.25 (1.25, 1.26) | 4.50 (4.42, 4.58) | 0.62 (0.61, 0.62) | 5.31 (5.18, 5.45) | 2.37 (2.32, 2.42) | 3.29 (3.21, 3.36) | 1.18 (1.16, 1.20) | 10.35 (10.01, 10.70) | 6.93 (6.70, 7.18) | 27.60 (24.20, 31.48) | 4.43 (4.13, 4.75) | 0.02 (0.02, 0.02) | 0.89 (0.82, 0.96) |
|  | 51-64 vs. <1 year | 1.77 (1.76, 1.78) | 5.68 (5.58, 5.79) | 0.57 (0.57, 0.58) | 5.15 (5.02, 5.28) | 1.59 (1.55, 1.62) | 2.88 (2.81, 2.94) | 0.78 (0.76, 0.79) | 12.38 (11.97, 12.80) | 6.22 (6.01, 6.44) | 23.56 (20.66, 26.87) | 2.83 (2.64, 3.03) | 0.01 (0.01, 0.01) | 0.95 (0.88, 1.03) |
| **Time period** | COVID-19 year 1 vs. pre-COVID-19 | 0.68 (0.68, 0.68) | 0.70 (0.70, 0.71) | 0.84 (0.84, 0.85) | 0.76 (0.76, 0.76) | 0.86 (0.86, 0.87) | 0.86 (0.85, 0.86) | 0.85 (0.85, 0.86) | 0.77 (0.77, 0.78) | 0.89 (0.89, 0.90) | 0.78 (0.77, 0.79) | 0.84 (0.83, 0.85) | 0.91 (0.85, 0.97) | 0.64 (0.62, 0.66) |
|  | COVID-19 year 2 vs. pre-COVID-19 | 0.66 (0.66, 0.66) | 0.69 (0.68, 0.69) | 0.86 (0.86, 0.86) | 0.76 (0.76, 0.76) | 0.93 (0.92, 0.93) | 0.90 (0.89, 0.90) | 0.89 (0.89, 0.90) | 0.79 (0.79, 0.80) | 0.88 (0.87, 0.88) | 0.80 (0.79, 0.80) | 1.04 (1.03, 1.06) | 1.10 (1.03, 1.16) | 0.36 (0.35, 0.38) |
|  | COVID-19 year 3 vs. pre-COVID-19 | 0.61 (0.61, 0.61) | 0.65 (0.65, 0.65) | 0.87 (0.87, 0.88) | 0.77 (0.76, 0.77) | 0.92 (0.91, 0.92) | 0.92 (0.91, 0.92) | 0.88 (0.88, 0.89) | 0.76 (0.76, 0.77) | 0.86 (0.85, 0.86) | 0.76 (0.76, 0.77) | 1.15 (1.14, 1.17) | 1.28 (1.21, 1.35) | 0.39 (0.37, 0.40) |
| **Sponsor's rank** | Senior enlisted vs. Junior enlisted | 0.70 (0.69, 0.70) | 0.71 (0.71, 0.71) | 0.69 (0.69, 0.69) | 0.54 (0.54, 0.54) | 0.64 (0.64, 0.64) | 0.62 (0.62, 0.63) | 0.65 (0.65, 0.66) | 0.59 (0.59, 0.60) | 0.63 (0.62, 0.63) | 0.92 (0.91, 0.93) | 1.01 (1.00, 1.02) | 1.12 (1.08, 1.17) | 1.36 (1.32, 1.41) |
|  | Officer vs. Junior enlisted | 0.96 (0.95, 0.96) | 1.06 (1.06, 1.06) | 1.20 (1.19, 1.20) | 0.83 (0.83, 0.83) | 1.03 (1.03, 1.04) | 1.01 (1.01, 1.02) | 1.04 (1.04, 1.05) | 0.84 (0.84, 0.84) | 1.08 (1.07, 1.08) | 1.05 (1.04, 1.06) | 1.07 (1.05, 1.08) | 1.90 (1.82, 1.99) | 1.31 (1.27, 1.36) |
| **Geographic region** | OCONUS vs. North | 1.45 (1.44, 1.45) | 1.59 (1.58, 1.60) | 1.67 (1.65, 1.68) | 1.42 (1.41, 1.43) | 1.49 (1.47, 1.50) | 1.30 (1.29, 1.32) | 1.11 (1.09, 1.12) | 1.25 (1.24, 1.26) | 0.81 (0.80, 0.82) | 0.68 (0.67, 0.70) | 0.69 (0.67, 0.71) | 0.37 (0.33, 0.43) | 0.53 (0.49, 0.57) |
|  | South vs. North | 1.11 (1.10, 1.11) | 1.24 (1.24, 1.24) | 1.06 (1.06, 1.07) | 1.05 (1.05, 1.06) | 1.05 (1.04, 1.05) | 1.03 (1.03, 1.04) | 0.99 (0.99, 1.00) | 1.04 (1.04, 1.05) | 0.95 (0.95, 0.96) | 0.92 (0.91, 0.93) | 1.10 (1.09, 1.12) | 0.91 (0.87, 0.96) | 1.20 (1.16, 1.23) |
|  | West vs. North | 0.94 (0.94, 0.94) | 0.95 (0.95, 0.96) | 1.03 (1.02, 1.03) | 1.07 (1.07, 1.08) | 1.03 (1.03, 1.04) | 0.99 (0.99, 1.00) | 0.94 (0.93, 0.94) | 0.92 (0.92, 0.93) | 0.79 (0.79, 0.80) | 0.78 (0.77, 0.79) | 1.13 (1.11, 1.14) | 0.90 (0.86, 0.95) | 0.90 (0.87, 0.93) |
| **Sex** | Female vs. Male | 0.41 (0.41, 0.41) | 0.46 (0.46, 0.47) | 0.85 (0.85, 0.86) | 0.73 (0.73, 0.74) | 1.23 (1.22, 1.23) | 0.92 (0.92, 0.93) | 1.33 (1.33, 1.34) | 0.65 (0.65, 0.65) | 0.92 (0.92, 0.93) | 1.34 (1.33, 1.35) | 1.64 (1.62, 1.66) | 1.08 (1.04, 1.13) | 1.40 (1.37, 1.44) |

*All Incident rate ratios are shown with a Bonferroni correction except for the umbrella category, labeled “any allergic conditions.”*
